# Monobloc implants in cementless total hip arthroplasty in patients with Legg-Calve-Perthes disease: a long-term follow-up

**DOI:** 10.1186/s12891-017-1748-1

**Published:** 2017-09-05

**Authors:** Ze-Yu Luo, Hao-Yang Wang, Duan Wang, Hui Pan, Fu-Xing Pei, Zong-Ke Zhou

**Affiliations:** 1 Department of Orthopedics, West China Hospital/West China School of Medicine, Sichuan University, 37# Wuhou Guoxue Road, Chengdu, 610041 People’s Republic of China; 2 Department of Hematology, The First Affiliated Hospital of Chongqing Medical University, No. 1 Youyi Road, Yuzhong District, Chongqing, 400016 People’s Republic of China

**Keywords:** Legg-calve-Perthes disease, Monobloc stem, Hip, Arthroplasty

## Abstract

**Background:**

The purpose of this study was to evaluate 10-year outcomes in cementless monobloc total hip arthroplasty (THA) in a group of hips with Legg-Calve-Perthes disease (LCPD).

**Methods:**

We reviewed 71 patients (88 hips) who underwent cementless THA with a diagnosis of LCPD from 2003 to 2009. From the total of 71 patients, 34 men and 37 women with an average age of 49.94 years were included. The mean follow-up period was 10 years.

**Results:**

The mean Harris Hip Score improved significantly from 46.42 to 89.70. Similarly, the postoperative range of motion, hip dysfunction and osteoarthritis outcome score and SF-12 score also significantly improved. The mean leg lengthening was 22.1 mm. During the follow-up, eight complications were noted, including two cases of intraoperative femoral fractures, two cases of sciatic nerve paralysis, two cases of heterotrophic ossifications, one case of thigh pain and one case of dislocation. One revision was conducted for a periprosthetic fracture, and the survivorship at 10 years was 98.3%.

**Conclusions:**

These data suggest that the monobloc stem can lead to satisfactory outcomes for clinical function, radiological evaluation, restoration of the normal limb lengths, complications, and survivorship among LCPD patients undergoing total hip arthroplasty.

## Background

Legg-Calve-Perthes disease(LCPD) is characterized by osteonecrosis of the femoral head during childhood [1, 2]. Similar to other childhood hip disorders, LCPD has the development of degenerative coxarthrosis as its natural history [3]. Total hip arthroplasty (THA) is recommended for patients with end-stage hip disease [4]. Nevertheless, there are limited data available in the literature regarding the characteristics and complications of THA in patients with a history of LCPD [5–13].

THA for patients with LCPD is known to be technically demanding because of the flattens and widens of the femoral head, excessive anteversion of the femoral neck, a straight and narrow medullary canal of the femur, a shallow and retroverted acetabulum, and the abnormality caused by previous operations [5]. Coxa breva, a typical residual deformity of LCPD, consists of a short femoral neck, a large oval-shaped femoral head, a relatively overgrown greater trochanter, and a decreased femoral neck-shaft angle [3]. The acetabulum is also deformed, becomes flat, and loses its concavity to accommodate the deformed femoral head. Frequently, the femoral head is subluxated laterally and incompletely covered by the acetabulum [3]. In 1984, the S-ROM system (DePuy, Warsaw, IN) was developed as a stem for patients with these various types of anatomic deformities. This stem has a modular mechanism with a high degree of freedom. It consists of two parts, the sleeve and stem body. In addition, the stem and sleeve have various combinations, and independent reaming in the proximal metaphyseal region of the femur and the diaphyseal region enables robust fixation with respect to various intramedullary canal shapes. Using the S-ROM prosthesis to treat LCPD has been reported to provide excellent results [8]. However, in with respect to modular femoral components, some risk factors need to be considered, such as fretting, corrosion, and mechanical failure, which may affect the stability of the stem and result in osteolysis and loosening. A recent series has documented the eight-year outcomes of 68 THAs with the use of a monobloc stem for LCPD [5]. However, monobloc cementless stems for patients with a history of Perthes’ disease raises several concerns: an increased risk of intraoperative femoral fracture, excessive anteversion of the femoral stem, malposition of the acetabular component, an increased risk of dislocation, unsatisfactory clinical and radiological results, and poor survivorship [5].

To address these concerns, we conducted this study to evaluate the 10-year outcomes of cementless monobloc THAs in a group of consecutive hips with LCPD.

## Methods

This was a retrospective study conducted at West China Hospital, Sichuan University, which serves as a tertiary level center in China. The retrospective study protocol was approved by the Institutional Review Board of West China Hospital, and informed consent was obtained from all of the patients. Between June 2003 and December 2009, a total of 82 consecutive patients who underwent THA with a diagnosis of LCPD were included in our study. Eleven patients were excluded from the study for different reasons. Six patients were lost to follow-up after surgery and could not be contacted due to incorrect telephone numbers and addresses. Three patients with incomplete chart records were excluded. Two patients died of chronic medical problems which were unrelated to the THA. Therefore, 71 patients (88 hips) were included. The clinical data of our patients were evaluated retrospectively after receiving approval from the Institutional Review Board of West China Hospital.

### Clinical data

There were 34 men and 37 women with an average age of 49.94 years (range, 25–73), and the average body-mass index (BMI) was 23.64 kg/m^2^ (range, 15.8–30.9). At our institution, patients undergoing arthroplasty are followed-up prospectively at regular intervals (at 3 weeks, 6 weeks, 12 weeks, and 6 months after surgery and annually thereafter). The time to follow-up is calculated as the time from surgery to the most recent visit. A clinical evaluation was conducted by two independent observers (ZY-L and HY-W) with the use of the Harris Hip Score (HHS), hip dysfunction and osteoarthritis outcome score (covering pain, symptoms, daily living, sports and recreational activities, and quality of life) and the SF-12 scale (physical component summary and mental component summary). The preoperative or postoperative hip range of motion (ROM) and visual analog scale for pain or satisfaction (VAS, pain/satisfaction) were also recorded. All complications were noted.

### Surgical technique

Before the operation, to restore the anatomical hip rotation center for monobloc prosthesis, an evaluation of the abnormal anatomy of the proximal femur and acetabulum, soft-tissue contracture, leg-length discrepancy and previous operations were routinely conducted. In patients with LCPD, the pelvis should be evaluated with special care to determine the amount of bone stock present for fixation of the cup. A three-dimensional CT scan is also helpful in evaluating the acetabulum. The width of the medullary canal is also noted because it may be narrow, especially in patients with LCPD. In these instances, a careful templating before surgery should be conducted. Templating aids in selecting the size of the implant that would restore the center of rotation of the hip and provide the best femoral fit.

All operations were performed via a posterolateral approach with the patient in the lateral decubitus position. In cases with remarkable LLD with coxa breva deformity, neck cutting was performed at the middle level of the femoral head, which is a more proximal location than that used in usual cases of THA for the restoration of LLD. The target position of the acetabular component was 40° to 45° abduction and 20° anteversion [5]. From small to large diameter, acetabular reaming was performed. The porous-coated acetabular component (DePuy, Pinnacle, Warsaw, IN) was inserted in the acetabular position with the use of a press-fit technique and fixed. Two screws were used to improve acetabular cup stability in one hip. The mean outer diameter of the acetabular cup was 52 mm (range 46 mm–58 mm). The mean head diameters were 34 mm (range 28 mm–36 mm), and three hips were 28 mm, 45 hips were 32 mm, and 40 hips were 36 mm. A ceramic-on-ceramic bearing was used in 78 hips (89%), a metal-on-polyethylene bearing was used in two hips (2%), and a ceramic-on-polyethylene bearing was used in eight hips (9%). After insertion of the acetabular cup, we focused on the femoral preparation. When preparing the femoral canal, restoration of the appropriate anteversion of the femur is necessary. The broach is aligned to match precisely the axis of the patient’s femoral neck. It is important to not attempt to place the broach in additional anteversion because this would lead to under sizing of the stem and insufficient rotational stability. The monobloc stems were inserted along the endosteal geometry of the proximal femur without manipulation to adjust the stem version, and a Corail stem (DePuy) was conducted in 61 hips and a Trilock stem (DePuy) was inserted in the other hips. Femoral fractures were defined and treated according to the Vancouver classification [14].

All of the patients had an antibiotic prophylaxis with a third-generation cephalosporin for a day. Low-molecular-weight heparin (0.2 mL, or 2000 AxaIU, of Clexane [enoxaparin sodium]) was first administered 8 h after surgery and then every 24 h until hospital discharge. The patients were allowed full weight-bearing on the day after surgery.

### Radiographic analysis

Radiographic evaluations were performed by two independent observers (ZY-L and HY-W) preoperatively and every time of follow-up with use of anteroposterior and lateral radiographs of the affected hip. The degree of deformity was defined by the classification system of Stulberg et al. [15] (Table 1). For determining the leg-length discrepancy, a line between the inferior edge of the acetabular teardrop (inter-teardrop line) can be used as the reference line. Perpendicular measurements to the tip of the greater trochanter are compared to compute the leg-length discrepancy. A radiographic evaluation was conducted by assessing the cup and femoral stem orientation and osteolysis at the times of follow-up.

**Table 1:** The Criteria Define of Stulberg Classifications

Classification	Criteria Define
Class I	A completely normal hip joint
Class II	A spherical femoral head (same concentric circle on anteroposterior and frog-leg lateral radiographs), but with one or more of the following abnormal characteristics of the femoral head, neck, or acetabulum:
	(1) Larger than normal (although spherical) femoral head (coxa magna);
	(2) Shorter-than-normal femoral neck;
	(3) Abnormally steep acetabulum
Class III	A non-spherical (ovoid, mushroom-shaped, or umbrella-shaped) but not flat femoral head. Abnormal characteristics of the femoral head, neck, and acetabulum (as described for Class II) are present also.
Class IV	A flat femoral head and abnormalities of the femoral head, femoral neck, and acetabulum
Class V	A flat femoral head and a normal femoral neck and normal acetabulum

**Fig. 1:**
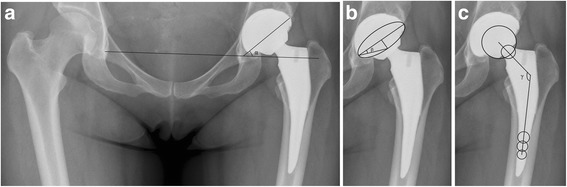
The cup orientation and stem version were measured in an anteroposterior pelvic radiograph. **a** Postoperative acetabular inclination: α. **b** Postoperative acetabular anteversion: β. **c** The angle between the axes of the neck and stem was regarded as the neck–shaft angle: γ. The stem version was assessed by the γ

The cup orientation was measured on the postoperative radiograph [8, 16]. While measuring the cup orientation on the anteroposterior radiographs, inclination is the angle between the face of the cup and the transverse axis (the inter-teardrop line); cup anteversion is calculated from the relative size of the major and minor diameters of the ellipse [16] (Fig. 1). Osteolysis around the cup was defined as a scalloped erosion exceeding 2 mm in diameter at the bone-prosthesis interface; the progressive widening of radiolucent lines >2 mm, migration of >2 mm, or 5° of cup tilting were defined as cup loosening [17]. Seven zones around the femoral component were described by Gruen et al. [18]. Anteversion of the femoral stem was calculated using the method described by Weber et al. [19] (Fig. 1). Stem fixation was classified as bony ingrowth and fibrously stable or being unstable according to the Engh classification [20–22]. Subsidence of the femoral component was defined by the method described by Loudon et al. [23]. Heterotopic ossification was graded according to Brooker et al. [24].

### Statistical analysis

We assessed the means and standard deviations for quantitative data, the frequencies, and the percentages for the qualitative data. The continuous variables were compared with independent exponent t- tests. The Pearson chi-square test or Fisher exact test was used to analyze the qualitative comparative parameters. Kaplan-Meier was utilized in the analysis of survivorship with the end points as a revision for any component. All of the data analyses were performed using SPSS for Windows, Version 19.0 (IBM Corp, Armonk, NY, USA). The significance was set at *P* < 0.05.

## Results

### Clinical data

The mean duration of the follow-up was 10.09 ± 1.84 years (range 7.5–13.9), as shown in Table 2. The outcomes regarding hip function are listed in Table 3. The mean HHS improved significantly from 46.42 ± 6.11 points (range 35–56) preoperatively to 89.70 ± 5.46 points (range 77–97) postoperatively. For the range of motion (ROM), the flexion improved from 90.13 ± 11.24° (range 30°-110°) preoperatively to 120.32 ± 4.94° (range 102°-125°), and the abduction improved from 29.28 ± 5.50° (range 10°-40°) preoperatively to 41.26 ± 3.73° (range 30°-45°). In addition, the hip dysfunction and osteoarthritis outcome score and the SF-12 score improved postoperatively compared with those preoperatively, and this difference was significant (Table 3).

**Table 2:** Baseline Characteristics, Radiographic Information, and Complications

Variables	Patients (*N* = 71)
Age, mean ± SD	49.94 ± 11.40
Gender[no.[%]of patients]	
Male	34(47.89%)
Female	37(52.11%)
Height(cm),mean ± SD	159.60 ± 6.59
Weight(Kg),mean ± SD	60.91 ± 11.54
BMI,mean ± SD	23.64 ± 3.62
Followup(year),mean ± SD	10.09 ± 1.84
Bilateral or unilateral[no.[%]of patients]	
Bilateral	17(23.94%)
Unilateral	54(76.06%)
Side[no.[%]of hips]	
Left	41(46.59%)
Right	47(53.41%)
Radiographic Information,mean ± SD	
Cup anteversion, °	25.95 ± 6.00
Cup abduction, °	39.01 ± 5.51
Femoral stem version, °	13.63 ± 4.10
Stulberg classifications [no.[%]of hips]	
Class II	1(1.14%)
Class III	30(34.09%)
Class IV	55(62.5%)
Class V	2(2.27%)
VAS Satisfaction, mean ± SD	9.82 ± 0.52
10	62(87.32%)
9	6(8.45%)
8	2(2.82%)
7	1(1.41%)
Complications[no.[%]of hips]	
Intraoperative femoral fracture	2(2.27%)
Postoperative temporary sciatic nerve paralysis	2(2.27%)
Heterotopic ossification	2(2.27%)
Thigh pain	1(1.14%)
Postoperative dislocation	1(1.14%)
Aseptic loosening	0
Deep vein thrombosis	0
Calf muscular venous thrombosis	0
Infection	0

*BMI* body mass index, *ONFH* Osteonecrosis of Femoral Head, *ASA* American Society of Anesthesiologists, *VAS* Visual Analogue Scale

**Table 3:** Outcomes Regarding Hip Function

Variables, mean ± SD	Patients (N = 71)		*P*
	Preoperative	Postoperative	
HHS			
Mean in points	46.42 ± 6.11	89.70 ± 5.46	0.01
Rating [no.[%]of patients]			
Excellent (90–100 points)	0	45(64.38%)	
Good (80–89 points)	0	23(32.39%)	
Fair (70–79 points)	0	3(4.23%)	
Poor (<70)	71(100%)	0	
VAS Pain	5.11 ± 1.30	0.30 ± 0.54	0.01
ROM			
Flexion	90.13 ± 11.24	120.32 ± 4.94	0.01
Abduction	29.28 ± 5.50	41.26 ± 3.73	0.01
HOOS			
Symptoms	11.37 ± 2.40	17.19 ± 1.45	0.01
Pain	19.39 ± 5.90	37.32 ± 2.05	0.01
Daily living	34.80 ± 3.78	65.77 ± 1.17	0.01
Sports and recreational activities	8.07 ± 1.77	14.55 ± 1.96	0.01
Quality of life	6.62 ± 1.21	13.42 ± 1.31	0.01
SF-12			
PCS	15.80 ± 2.69	23.11 ± 2.78	0.01
MCS	16.63 ± 2.80	24.55 ± 3.13	0.01
Limp [no.[%]of patients]			0.01
Severe	0	0	
Moderate	34(47.89%)	0	
Slight	20(28.17%)	3(4.23%)	
None	17(23.94%)	68(95.77%)	
Limb length discrepancy			
Mean in mm	24.3 ± 7.8	2.4 ± 2.8	0.01
Rating [no.[%]of hips]			
< 10	5(5.68%)	87(98.86%)	
10–20	21(23.86%)	1(1.14%)	
20–30	36(40.91%)	0	
30–40	26(29.55%)	0	

*HHS* Harris Hip Score, *VAS* Visual Analogue Scale, *HOOS* Hip dysfunction and Osteoarthritis Outcome Score, *ROM* range of motion; SF-12: 12-item short-form health survey questionnaire, *PCS* physical component summary, *MCS* mental component summary

### Radiographic outcomes

The mean cup anteversion and abduction were 25.95 ± 6.00° (range 15°-36°) and 39.01 ± 5.51° (range 27°-50°), respectively. The mean stem anteversion was 13.63 ± 4.10° (range 5° to 27°). Preoperative and postoperative LLD were 24.3 ± 7.8 mm (range 8 mm–36 mm) and 2.4 ± 2.8 mm (range − 2 mm-9 mm), respectively. The mean leg lengthening was 22.1 ± 7.8 mm (range 4 mm–36 mm) postoperatively. Although no cases with >10 mm of LLD were identified, three slightly limp patients were noted by the final follow-up.

**Fig. 2:**
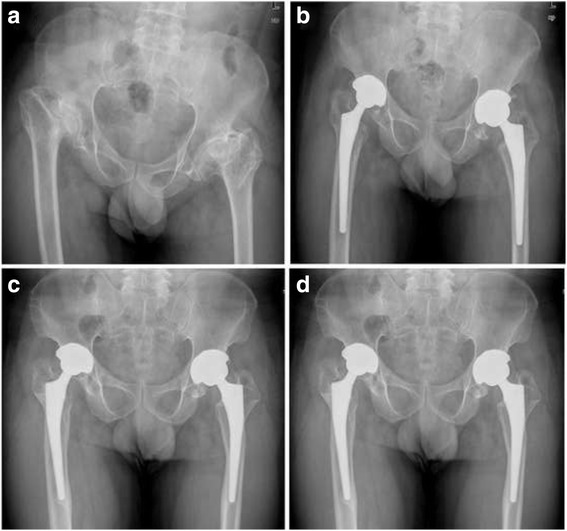
The radiographs illustrated a 41-year-old man with bilateral Legg-Calve-Perthes disease (LCPD) treated by total hip arthroplasty (THA) with the monobloc stem. **a** Preoperative anteroposterior view. **b**: Postoperative radiographic image. The hip was reconstructed at the level of the anatomic hip center by total hip arthroplasty. No complication occurred during the operation. **c** At 4-year follow-up, no radiolucent lines were found. **d** At the 11-year follow-up, no migration, osteolysis, or subsidence was detected. The femoral and acetabular components were considered stable

No case noted radiolucent lines or migration around the acetabular cup. For the stems, we did not note a loosening or subsidence at the latest follow-up. One hip was detected with a radiolucent line around the proximal femoral, which was not progressed during the follow-up. Other femoral stems achieved either bony ingrowth or and fibrous ingrowth at the final follow-up (Fig. 2). Peri-articular heterotrophic ossification was present in two hips and each of the two cases was defined as class II with Brooker classification system and didn’t require excision before the final follow-up.

### Complications

Of the 88 hips, seven hips sustained a total of 8 complications (Table 2). Two cases experienced an intraoperative femoral fracture. All of the three cases were type A2 according to the Vancouver classification [14] were cured with cerclage cable. Two patients sustained a sciatic nerve paralysis with a leg lengthening of 31 mm and 36 mm. Both were resolved at the final follow-up. One patient complained of thigh pain at the last follow-up. One patient presented a dislocation at the first day after the operation and was treated with a closed reduction with no further sequelae. No evidence of deep venous thrombosis, calf muscular venous thrombosis and infection were noted during the follow-up.

### Survivorship analysis

**Fig. 3:**
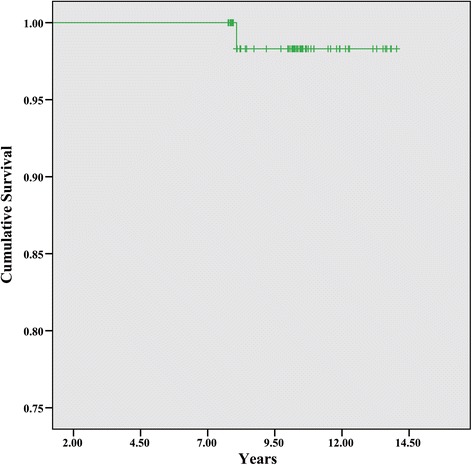
A Kaplan-Meier survivorship curve with any revision for any component as the end points

During the follow-up period, one revision was conducted for a periprosthetic fracture at 8 years. Using revision for any reason as an end point, the Kaplan-Meier survival estimate at 10 years was 98.3% (95% confidence interval [CI], 94.9%–99.9%) (Fig. 3).

## Discussion

THA in a patient with Legg-Calve-Perthes disease can be a technically challenging procedure because of the high complication and the complex features of the bone, soft tissue contractures and the relatively young age of the patients [10]. The prior studies validated the benefits of THA in patients with severe arthritis associated with LCPD [5–13] (Table 4). However, most prior reports of LCPD included mixed groups of different types of components in a small number of patients [6, 10, 12]. To our knowledge, this is the largest reported series in which the results of a monobloc stem for the treatment of LCPD were evaluated, including (1) complications; (2) clinical and radiographic outcomes; and (3) survivorship.

**Table 4:** Overview of Relevant Literature in the Treatment of Legg–Calve–Perthes Disease with Total Hip Arthroplasty

Study	Year	Patient (Hips)	Mean Age(y)	Mean FollowUp (yrs)	Implantation Type	Preoperative LLD(mm)	Postoperative LLD(mm)	Preoperative Function	Postoperative Function	Revision	Complication	Survival
Lee et al.	2017	68 (68)	48(16–73)	8.5(5.2–10)	Monobloc Cementless[Corail, DePuy]	NA	2.2(−2–14)	NA	HHS: 91 (73–100) UCLA activity: 5.5 (3–8)	None	Sciatic nerve palsy: 1 Ectopic ossification: 9	100%
Huang et al.	2016	33(33)	46.8 (24–72)	4.5 (2.2–11.3)	Various: Monobloc cementless: 22 Modular Femoral Component[SROM, DePuy]: 11	NA	NA	HHS: 37.6 (15–56)	HHS: 92.8(70–100) Diminished or no limping: 45% (15/33) dissatisfied: 9.1%(3/33)	None	Ectopic ossification: 2 Sciatic nerve palsy: 1 Intraoperative fracture: 4	NA
Lee et al.	2016	37(41)	43.8 ± 12.7 (18.9–66.3)	10.4 ± 3.3 (5.2–16.0)	Aluminae-alumina Cementless	20 ± 12	2 ± 9	HHS:66.7 ± 17.4	HHS: 96.8 ± 6.8 Lengthening: 17 ± 9 Diminished or no limping: 97% (36/37)	None	Intraoperative fracture: 14 Peroneal nerve palsy: 1 Deep vein thrombosis: 1	NA
Seufert et al.	2015	28(35)	51.6 (22–74)	8.2 (2–14)	Modular Femoral Component[SROM, DePuy]	18.6	7	HHS: 49.8 ± 9.2	HHS: 93.9 ± 8.5 Diminished or no limping: 94.3% (33/35)	Deep Infection: 1	Ectopic ossification: 2 Dislocation: 1	NA
Lim et al.	2014	23(23)	49.2 (25–69)	3.5 (2–7)	Monobloc Cementless: Bencox II 22,Accolade 1	15 (7–25)	3 (1–7)	HHS: 48.3 (42–67)	HHS: 92.4 (79–97)	Sciatic nerve palsy: 1	Sciatic nerve palsy: 1 Intraoperative fracture: 3	96%
Al-Khateeb et al.	2014	14(15)	32.8 (23–55)	10.1 (5–15)	Custom-made Cementless	NA	NA	HHS: 41(27–57) Trendelenburg sign(+): 11(78%) Walking aids: 8	HHS: 80(51–94) Trendelenburg sign(+): 3(21%) Walking aids: 1	Aseptic loosening: 3 Deep infection: 1	Ectopic ossification: 1 Dislocation: 1	Femoral stem: 100% Acetabular components: 79%
Baghdadi et al.	2013	95(99)	48 ± 15	8 ± 5	Cementless implants: 76 Hybrid implants:21	NA	NA	HHS: 56 (25–85)	HHS: 88 (50–100)	Aseptic loose stem component: 5 Liner wear: 3 Aseptic loose cup component: 1 Deep infection: 1	Intraoperative fracture: 8 (femoral), 1 (acetabular) Sciatic nerve palsy: 3 Thromboembolic: 1 Infection: 1 Dislocation: 1 Adductor tenotomy: 1	Cementless implants:90% (76%–96%) Hybrid implants:86% (57%–96%)
Traina et al.	2011	27(32)	37.8(19–65)	12.3(5–21)	Cement-less: 31 Cemented:1	NA	9(0–26)	HHS: 50.1(25–75)	HHS: 87.5 (73–96) Average Lengthening: 12 (6–27)	Failure for cemented component: 1	Sciatic nerve palsy: 2 Intraoperative fracture: 1 Hematoma: 1	96.9%(90.8% -100.0%)
Pietrzak et al.	2011	9(9)	56(41–69)	15(11–21)	Cement-less: 6 Cemented:3	NA	NA	HHS: 34 WOMAC: 77	HHS: 93 WOMAC: 6	None	NA	100%
Current study	2017	71(88)	49.94 ± 11.40(36–73)	10.09 ± 1.84(7.7–14)	Monobloc cementless: 88	24.3 ± 7.8(8–36)	2.4 ± 2.8(−2–10)	HHS: 46.42 ± 6.11, Sym: 11.37 ± 2.40, Pain:19.39 ± 5.90, DL: 34.80 ± 3.78, SARA: 8.07 ± 1.77, QOF: 6.62 ± 1.21, PCS: 15.80 ± 2.69, MCS: 16.63 ± 2.80	HHS: 89.70 ± 5.46, Sym: 17.19 ± 1.45, Pain:37.32 ± 2.05, DL: 65.77 ± 1.17, SARA: 14.55 ± 1.96, QOF: 13.42 ± 1.31, PCS: 23.11 ± 2.78, MCS: 24.55 ± 3.13	Fracture: 1	Intraoperativel fracture: 2 Sciatic nerve palsy:2 Heterotopic ossification: 2 Dislocation: 1 Thigh pain: 1	98.3%(94.9%–99.9%)

*LLD* Limb length discrepancy, *HHS* Harris Hip Score, *UCLA* University of California activity, Los Angeles activity scales, *WOMAC* The Western Ontario and McMaster Universities Osteoarthritis Index, *Sym* Symptoms, *DL* Daily living, *SARA* Sports and recreational activities, *QOF* Quality of life, *PCS* physical component summary, *MCS* mental component summary, *NA* not applicable

LCPD is osteonecrosis of the juvenile hip, which was first described by Arthur Thornton Legg in 1909 as “An Obscure Affection of the Hip Joint”, shortly after Roentgen technology was discovered in 1895. Some longitudinal studies have also shown that in some cases, Legg-Calve-Perthes disease that had begun as a purely femoral abnormality (producing a pistol grip deformity) could then secondarily lead to acetabular deformity [15]. Although the acetabulum can accommodate a deformed femoral head and restore what may seem like reasonable congruence on an AP radiograph, this cannot be achieved for all movements of the hip. This results in an ‘incongruous incongruity’, which could lead to subsequent osteoarthritis [25].

Resurfacing arthroplasty for LCPD is an alternative to conventional THA. Two authors have advocated the use of hip resurfacing for patients with LCPD because of the usually young age and high activity level of these patients [26, 27]. Although, it has advantages of preservation of the proximal femur, a wider range of motion, and low wear rate of its metal-on-metal bearing surface, it is difficult to gain leg length, and the greater trochanter causes impingement. In addition, the thick mantle cement is also known to be a possible cause of early failure [3].

The LCPD patient group has well-known negative prognostic factors for THA, such as young age and abnormality. The outcomes of the present series demonstrate that standard total hip replacement with a monobloc stem can be a feasible option for patients with osteoarthritis of the hip secondary to LCPD.

Increased anteversion of the femoral neck is a common finding for LCPD [28]. Accordingly, the stem may be excessively anteverted and may result in dislocation. Therefore, choosing the femoral component is rather important. For this reason, custom-made versions have been preferred. Al-Khateeb reviewed 15 THAs with an average follow-up of 10 years [10]. Although, only one patient sustained a dislocation and was treated with a closed reduction with no further sequelae, the amount of revision arthroplasty was 21%. In addition, custom-made stems were rather expensive. Seufert et al. preferred the use of modular stem for the appropriate contact and restoration of appropriate anteversion [8]. However, concerns over taper fretting, corrosion and even stem fracture with modular stems continue to be important factors when choosing this type of stem for a patient. Lee et al. assessed implant position and the medium-term results of monobloc cementless THA [5]. The mean stem version was 14.6° (range- 2.3° to 30°) with no hip dislocation. In the present study, the mean stem version was 13.63 ± 4.10° (range 5° to 27°), and one dislocation occurred. The dislocation was rare in Lee et al. and the present study, which demonstrated that the monobloc stem can achieve satisfactory results regarding the LCPD. However, Lee et al. only utilized the middle-length stem, and we chose both short and middle-length monobloc stem in this series, which could have affected the result.

Prior studies have demonstrated that standard total hip replacement could restore excellent clinical function for the LCPD patients [11, 12, 29]. In our series, we evaluated the clinical function of the patients with HHS, hip dysfunction and osteoarthritis outcome score (covering pain, symptoms, daily living, sports, and quality of life), the SF-12 scale (physical component summary and mental component summary) and the visual analog scale for pain or satisfaction (VAS, pain/satisfaction). We confirmed that the monobloc stem could improve hip function. In addition, the quality of life and mental condition also improved after the operation. Traina et al. demonstrated in a 10-year follow-up that the average Harris hip score improved from 50.1 to 87.5 for LCPD [12]. Seufert et al. confirmed that Harris hip scores, on average, improved from 49.8 (26–73) to 93.9 (82–100) (*P* < 0.05) after a minimum of 2 years of follow-up [8]. Thus, our series compares favorably with other reports of THA in LCPD patients.

In addition, postoperative complications require special attention for the LCPD patients. In the current study, seven hips sustained a total of 8 complications. For the femoral fracture, 2 (2%) cases occurred, which was a low rate compared with the fracture rate that has been reported to vary between 0 and 34.1% for cementless THA in LCPD [6, 8, 10]. Lee et al. demonstrated that a wedge-shaped stem with bulky proximal design seemed unsuitable for femurs with anatomical deformity due to the risk of intraoperative femoral fracture [6]. Seufert et al. recommended a modular prosthesis which could make appropriate contact of the metaphysis and the diaphysis. In the current study, a monobloc stem could be available for LCPD with 2 cases of fracture. Use of a monobloc stem for LCPD should be recommended and carefully planned during preoperative planning according to preoperative templating and the degree of anatomical deformity. Careful templating aids in selecting the type of implant that would restore the center of rotation of the hip, provide the best femoral fit and decrease the femoral fracture.

Nerve injury is another complication we need to carefully monitor. An analysis of the literature by Goetz et al. determined the risk of nerve palsy after primary total hip arthroplasty to be 0.5% for arthritis, 2.3% for hip dysplasia, and 3.5% for revision surgery [30]. In the current study, two patients (2%) sustained a sciatic nerve paralysis with a leg lengthening of 3.1 cm and 3.6 cm and all the two cases were resolved at the final follow-up. Baghdadi et al. detected three cases (3%) of sciatic nerve paralysis that were lengthened by 2, 1.3, and 3.2 cm (mean, 2.2 cm) at the time of THA compared with a mean of 1.4 ± 1 cm in the patients who did not sustain a neurologic injury (*P* = 0.3) [11]. In the prior study, the association between limb lengthening and sciatic nerve palsy has been studied with varying conclusions. Edwards et al. correlated the amount of lengthening with the development of sciatic palsy and complete sciatic palsy occurred with a lengthening of 4.0 to 5.1 cm [31]. In contrast, Eggli S et al. found nerve palsy had no correlation with the amount of lengthening, which was most commonly caused by direct or indirect mechanical trauma [32]. The cause of sciatic palsy is still not clear, and we believe the excessive lengthening and mechanical trauma can both be potential causes. For patients with LCPD with severe leg-length discrepancy, shortening osteotomies may be considered to avoid nerve injury. In addition, careful exposing, mobilizing, and protecting the nerve and avoiding a stretch injury or direct contusion of the nerve are needed.

Finally, one patient conducted a revision and the Kaplan-Meier survival estimate at 10 years was 98.3% (95% confidence interval [CI], 94.9%–99.9%). Traina et al. reported the THA survivorship in patients with a history of LCPD at a 96% survivorship at 15 years [12]. Seufert et al. reported one revision (3%) at 3 years with a modular stem in patients with a history of LCPD [8]. Lee et al. reported no revision was required during a 9–15 years of follow-up with a monobloc stem of LCPD [5]. From the results of the present and prior studies, the survivorship of the monobloc stem was shown to be satisfactory.

There are several limitations to our study. First, it was a retrospective review at a single center. Second, the number of patients was relatively small. However, our study group is the largest series of patients with such an uncommon diagnosis. Third, two kinds of monobloc stems and three combinations of bearing were used. Fourth, all the patients’ functional outcomes were based on conventional questionnaire-based outcome measures. It would be more objective and reliable if a quantitative gait analysis was conducted for the patient. Finally, all operations were performed by five senior surgeons, which may affect the validity of our findings. However, in this series, all of the chosen implants and the surgical techniques were decided by the five senior surgeons together for all THAs.

## Conclusion

These data suggest that the monobloc stem can lead to satisfactory outcomes of the clinical function, radiological evaluation, restoration of normal limb lengths, complications, and survivorship among LCPD patients undergoing total hip arthroplasty.

## Abbreviations

DL: Daily living
HHS: Harris Hip Score
HOOS: Hip dysfunction and osteoarthritis outcome score
LCPD: Legg-Calve-Perthes disease
LLD: Limb length discrepancy
MCS: Mental component summary
NA: Not applicable
PCS: Physical component summary
QOF: Quality of life
ROM: Range of motion
SARA: Sports and recreational activities
SF-12: 12-item short-form health survey questionnaire
Sym: Symptoms
THA: Total hip arthroplasty
UCLA: University of California activity, Los Angeles activity scales
VAS: Visual Analogue Scale
WOMAC: The Western Ontario and McMaster Universities Osteoarthritis Index
